# The Role of Reprogrammed Glucose Metabolism in Cancer

**DOI:** 10.3390/metabo13030345

**Published:** 2023-02-25

**Authors:** Meran Keshawa Ediriweera, Sharmila Jayasena

**Affiliations:** Department of Biochemistry and Molecular Biology, Faculty of Medicine, University of Colombo, Colombo 08, Sri Lanka

**Keywords:** glucose, cancer, cancer treatment, metabolism

## Abstract

Cancer cells reprogram their metabolism to meet biosynthetic needs and to adapt to various microenvironments. Accelerated glycolysis offers proliferative benefits for malignant cells by generating glycolytic products that move into branched pathways to synthesize proteins, fatty acids, nucleotides, and lipids. Notably, reprogrammed glucose metabolism and its associated events support the hallmark features of cancer such as sustained cell proliferation, hijacked apoptosis, invasion, metastasis, and angiogenesis. Overproduced enzymes involved in the committed steps of glycolysis (hexokinase, phosphofructokinase-1, and pyruvate kinase) are promising pharmacological targets for cancer therapeutics. In this review, we summarize the role of reprogrammed glucose metabolism in cancer cells and how it can be manipulated for anti-cancer strategies.

## 1. Introduction

**Figure 1:**
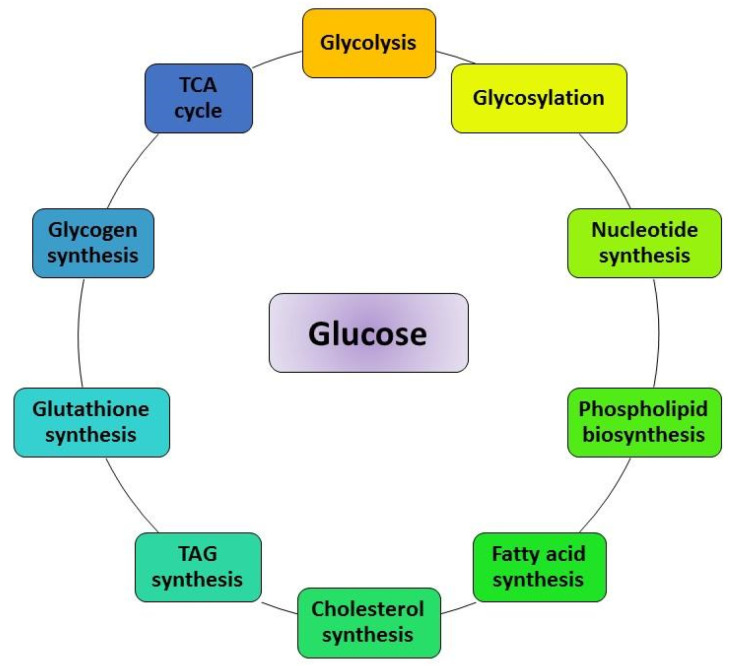
Biochemical fates of glucose. Glucose serves as a key molecule for fatty acid synthesis, cholesterol synthesis, TAG synthesis, glutathione synthesis, glycogen synthesis, phospholipid, and nucleotide synthesis.

Accelerated glycolysis and increased cellular uptake of glucose through intricate metabolic reprogramming are frequently observed in human cancers. Cells and tissues of multicellular organisms require a constant supply of various nutrients. The process of obtaining these nutrients in cells is normally subjected to strict genetic and hormonal control [1,2,3]. Glucose, a water-soluble six-carbon molecule, serves as one of the key molecules in the metabolism of animals (Figure 1). Apart from its role as a fuel, glucose provides a variety of metabolic intermediates for a wide range of biosynthetic reactions. The fundamental role of glucose metabolism, particularly glycolysis, in sustaining cell viability and growth is evident from its evolutionary conservation across all cellular forms of life. The breakdown of a glucose molecule into the 3-carbon pyruvate or lactate through glycolysis provides a key source of energy (ATP) and other substrates needed for cells. 

Glycolysis serves as the central hub of glucose catabolism, producing two molecules of pyruvate with energy stockpiled as ATP and NADH. In aerobic tissues, under aerobic conditions, pyruvate is produced following the complete degradation of glucose. Following oxidation, pyruvate produces acetyl-coenzyme A, which then undergoes complete oxidation by the citric acid cycle. In mitochondria, electrons donated by oxidation reactions are passed through a chain of electron transfer proteins. The electron transfer in mitochondria is associated with proton exchange across the mitochondrial membrane, giving rise to an electrical and chemical gradient, providing energetically favorable proton-motive forces for ATP synthesis. For each molecule of glucose metabolized to pyruvate, 2 ATP molecules are consumed in the preparatory phase and 4 ATP molecules are generated in the payoff phase in glycolysis, resulting in a net production of 2 ATP molecules per glucose molecule. However, the pyruvate generated possesses much energy that can be extracted by oxidative pathways in the TCA cycle compared to the energy preserved in ATP. 

In the absence of mitochondria or under anoxic conditions, pyruvate undergoes an additional step of forming lactate and is thus named ‘anaerobic glycolysis’. The conversion of pyruvate to lactate, under anaerobic conditions, provides the required reducing equivalents for the continuation of the glycolysis and is thus considered to be the primary reason for its formation, in an environment where glycolysis is the primary source of energy. However, this hypothesis has been challenged with the observation of much higher rates of glycolysis alongside predominant lactate formation despite an aerobic environment in malignant cells. 

Tumor cells preferentially carry out glycolysis, converting glucose to lactate at higher rates, in comparison to normal cells, despite the availability of oxygen: the so-called Warburg effect [4]. This can be considered the most significant consequence of the metabolic reprogramming that occurs during tumor formation. Although Warburg assumed frequent mitochondrial defects followed by accelerated glycolysis in cancer cells [5], mitochondrial defects are not always common in cancer cells, and mitochondrial and oxidative phosphorylation are essentially functioning in a variety of cancer cells [6]. 

In the initial stages of tumor formation, cells may indeed experience a hypoxic environment until a capillary bed has been formed. Additionally, many cells in the internal part of a tumor may not have access to the capillary network. As such, a majority of the cells in a tumor may have to depend on increased glycolysis for ATP production. This increased demand is met by increased expression of the glycolytic enzymes, at least partly activated through increased transcription via hypoxia-inducible factor 1 (HIF1), which is a critical regulator of many glycolytic enzymes and a key driving force that triggers abnormal glycolytic flux in malignant cells [7,8]. 

It has been observed that, apart from hypoxic conditions, several oncogenic signaling pathways activate HIF1, which has been identified to play a key role in the glycolytic switch in tumors [9]. The HIF1 is responsible for transcriptional activation of a majority of glycolytic enzymes while inhibiting the conversion of pyruvate to acetyl-CoA by activating pyruvate dehydrogenase kinase 1 (PDK1), which inactivates pyruvate dehydrogenase [10]. The prevention of pyruvate entry to the TCA cycle is therefore inhibited, pushing the pyruvate to form lactate. In addition to HIF1, the oncogene *Akt* named the ‘Warburg kinase’, drives various metabolic events in cancer cells [11]. 

Although oxidative phosphorylation (OXPHOS) generates ATP with higher efficiency at a slower rate than glycolysis does [12], the increase in the rate of glycolysis compared to mitochondrial oxidation implies that glycolysis serves more than just energy in cancer cells. Indeed, the metabolic intermediates of both glycolysis and the TCA cycle provide an array of metabolic intermediates required for cell growth and proliferation. For example, glucose-6-phosphate is shunted to the pentose phosphate pathway (PPP), which provides ribose sugars for nucleotide biosynthesis [13]. In this review article, we discuss the role of reprogrammed glucose metabolism in cancer and how it can be utilized for anti-cancer strategies. In addition, we discuss how glucose metabolism impacts cancer hallmarks in brief. Finally, we illustrate potential pharmacologically important intermediates of glycolysis that can be targeted with small molecules. 

## 2. Overview of Glycolysis 

**Figure 2:**
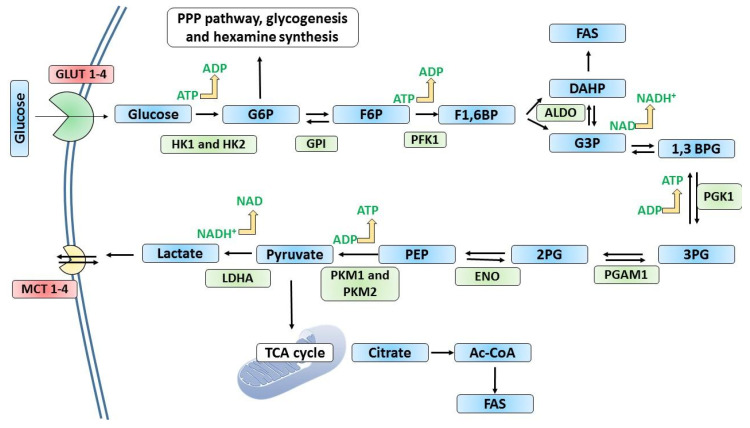
The steps involved in glycolysis. In glycolysis, a molecule of glucose gives two molecules of pyruvate through a series of enzymatically catalyzed reactions. ATP and NADH store some of the free energy released from glucose. Glycolysis comprises two main phases, namely the preparatory phase and the payoff phase. The first five stages of glycolysis, starting from glucose phosphorylation to the formation of G3P and DAHP, belong to the preparatory phase, while the payoff phase starts with the oxidation of G3P and ends with the formation of pyruvate. For each glucose molecule entering glycolysis, 2 ATP molecules are used in the preparatory phase and four ATP molecules are generated in the payoff phase, giving a net yield of 2 ATP molecules per glucose molecule entering glycolysis. Pyruvate formed in glycolysis has three possible catabolic fates. In aerobic conditions, pyruvate is completely oxidized by the TCA cycle and electrons donated by oxidation reactions are taken by oxygen (O_2_) through electron transfer reactions in mitochondria to generate water. The energy released by electron transfer reactions facilitate the synthesis of ATP. In anaerobic conditions, pyruvate is converted to lactate. In some microorganisms and plants, pyruvate is fermented to ethanol and carbon dioxide. 1,3 BPG; 1,3-bisphosphoglycerate, 2PG; 2-phosphoglycerate, 3PG; 2-phosphoglycerate, ALDO; aldolase, DAHP; dihydroxyacetone phosphate, ENO; enolase, F1,6BP; fructose-1,6-bisphosphate, F6P; fructose-6-phosphate, FAS; fatty acid synthesis, G3P; glyceraldehyde 3-phosphate, G6P; glucose 6 phosphate, GLUTs; glucose transporters, GPI; glucose-6-phosphate isomerase, HK1 and HK2; hexokinase 1 and 2; LDHA; lactate dehydrogenase, MCT; monocarboxylate transporters, PEP; phosphoenolpyruvate, PFK1; phosphofructokinase-1, PFKFB; 6-phosphofructo-2-kinase/fructose-2,6-bisphosphatase, PGAM1; phosphoglycerate mutase 1, PGK1; phosphoglycerate kinase 1, PKM1; pyruvate kinase 1 and PKM2; pyruvate kinase 2. Glycolytic enzymes are subjected to tight regulation in association with other metabolic pathways.

In glycolysis, as the name implies, the 6-carbon glucose molecule is ‘lysed’ or split into two to yield two 3-carbon products, pyruvate or lactate (Figure 2). The catabolism of glucose commences with its initial conversion to glucose-6-phosphate (G6P) by the action of hexokinase (HK), along with an investment of ATP as the donor of the phosphoryl group. This reaction plays a critical role in trapping the glucose within the cytoplasm and allows the continued transport/diffusion of glucose into the cells. 

The phosphorylated glucose is then prepared for splitting; first, the G6P is isomerized to fructose 6-phosphate (F6P) by the action of glucose-6-phosphate isomerase (GPI). Then, phosphofructokinase (PFK1) catalyzes the second phosphorylation in the pathway, forming fructose-1,6-bisphosphate (F16BP). This reaction requires the investment of a second ATP molecule. PFK1 is activated when cells are energy-depleted, indicated by a decreased ATP:ADP ratio and increased AMP. It is similarly inhibited when the cell has an adequate supply of ATP or other fuels. Additionally, PFK1 is regulated by several other allosteric regulators including fructose-2,6-bisphosphate (F2,6BP) and ribulose 5-phosphate. 

With phosphates added to carbons 1 and 6, the sugar is then split into two by fructose-1,6-bisphosphate aldolase (FBPA) or simply aldolase, to yield glyceraldehyde 3-phosphate and dihydroxyacetone phosphate. The dihydroxyacetone phosphate is reversibly converted to glyceraldehyde 3-phosphate by triose phosphate isomerase. In the subsequent steps, glyceraldehyde 3-phosphate is converted to 1,3-bisphosphoglycerate by glyceraldehyde 3-phosphate dehydrogenase (GAPDH), utilizing NAD^+^. The high-energy phosphoryl group at C1 is then transferred to ADP by phosphoglycerate kinase (named for its reverse reaction that occurs in gluconeogenesis)**,** generating the first ATP from glycolysis, and yields 3-phosphoglycerate (3PG). In the next step, the phosphoryl group remaining at C3 is transferred onto C2 by phosphoglycerate mutase, to form 2-phosphoglycerate (2PG). The next step generates a compound with a high phosphoryl group for transfer potential; enolase converts 2PG to phosphoenolpyruvate in a dehydration reaction. Then, in the final reaction of glycolysis, pyruvate kinase carries out the second substrate-level phosphorylation by transferring the phosphoryl group of phosphoenolpyruvate onto ADP, to yield pyruvate and ATP [14].

## 3. Metabolic Reprogramming in Cancer at the Rate-Limiting Steps of Glycolysis

The activities of glycolytic enzymes are subject to several negative and positive feedback regulatory mechanisms [15]. Malignant cells overcome these mechanisms to meet their anabolic demands and maintain biosynthetic needs by making use of them. Dysregulated or overproduced glycolytic enzymes drive tumorigenesis and are frequently seen in many types of human cancers. Cancer cells modulate the activity of three committed steps in glycolysis through several common molecular mechanisms to fulfill their anabolic requirements, thus providing a metabolic switch towards aerobic glycolysis [15]. 

In mammals, four major types of HKs are found, encoded by four different genes: *HK1*, *HK2*, *HK3*, and *HK4*. The first committed step of glycolysis, phosphorylation of glucose to yield (G6P), is catalyzed by HKs. This step facilitates glucose uptake into cells by creating a concentration gradient [16]. The activity of HKs 1–3, known as high-affinity HKs, is inhibited by an excess of catalytic product G6P, whereas the activity of HK4, a low-affinity hexokinase, is not inhibited by G6P, and it can therefore function when other HKs (1–3) are inhibited by G6P [17]. The expression pattern of different HK isozymes in the liver and muscle reveals different roles of these organs in glucose metabolism [14]. The expression of HK4 is predominant in the liver and pancreas and is also commonly referred to as glucokinase. HK1 and HK2 are localized at the outer mitochondria membrane and bound to voltage-dependent anion-selective channel 1 (VDAC1), obtaining mitochondrial ATP to phosphorylate glucose, which bridges OXPHO with glycolysis [17]. Compared to normal cells, cancer cells show elevated expression of HK2, which is responsible for the increased glucose flux to some extent in cancer cells, making HK2 an attractive pharmacological target for cancer therapy [18,19,20,21]. Mutant p53, which is abundantly found in cancer cells, can bind to the HK2 promoter and control its transcriptional activity [22]. The hypoxia-inducible factor HIF1-α binds to the hypoxia-responsive elements (HREs) in the HK2 promoter and increases its activity [23]. The methylation patterns of the *HK2* promoter influence its regulation and expression [24]. Autophagy has dual roles in cancer, both tumor promotion and suppression. Telomerase has been reported to regulate autophagy by activating HK2 expression through a telomerase-HK2-mTOR—autophagy pathway [25]. A recent investigation provides evidence that dysregulation of HK2 and mir-148a signaling is responsible for cisplatin resistance in cervical cancer cells, highlighting this signaling axis as a potential therapeutic target [26]. The MYC proto-oncogene has been reported to increase the expression of HK2 transcriptionally [27]. Long noncoding RNA (lncRNA) HOTAIR regulates the expression of HK2 by binding to miR-125 and miR-143 [28]. AKT aids the binding of HK1 and HK2 with mitochondria and facilitates their activity, which mediates mitochondrial protection [29,30]. AKT has been shown to directly phosphorylate HK2 at threonine (Thr-473), leading to a protective effect in cardiomyocytes [30]. Increased mitochondria-bound HK2activity exerts protective roles against oxidant-induced programmed cell death, implying that elevated activity of HK2 contributes to avoiding programmed cell death in cancer cells [31]. The roles of HK3 and HK4 in cancer glucose metabolism are not well-understood [15]. 

Phosphofructokinase 1 (PFK1), the second rate-limiting enzyme of glycolysis, catalyzes the conversion of fructose-6-phosphate (F6P) to fructose 1,6, bisphosphate (F1,6-BP). PFK1 is allosterically inhibited by high ATP levels and activated by fructose 2,6 bisphosphate. This powerful activator of PFK1 is formed by 6-phosphofructo-2-kinase/fructose-2,6-biphosphatase 3 (PFKFB, also known as PFK2) [14,32]. The two tandem domains of PFKFB possess kinase and phosphatase activity. The domain with kinase activity phosphorylates F6P to F2,6-BP, while the domain with phosphatase activity dephosphorylates F2,6BP to F6P [32,33]. The expression of PFKFB is ubiquitous and its four main isoforms (PFKFB1–4), encoded by four distinct genes, show different biological properties [34]. The glycolytic role of PFKFB in human cancer is well-described [35]. The isoenzyme encoded by the *pfkfb3* gene displays the highest kinase activity and is overexpressed in a range of cancer cells [36,37]. The activity of PFK1 is inhibited by several downstream products of glycolysis (high ATP levels, citrate, palmitoyl-CoA, and PEP), which facilitates glucose push into nucleotide synthesis through the pentose phosphate pathway, thereby generating adequate reducing potentials [15,33]. The expression of PFKFB3 is induced by HIF under hypoxic conditions [38,39]. In acute myeloid leukemia, mTOR acts as a stimulator of PFKFB3 [40]. PFK1, a key driver of glycolytic flux, possesses three isoforms namely, liver (PFKL), muscle (PFKM), and platelet (PFKP) [41]. PFKP is overexpressed in a range of human cancers [15,42]. The phosphorylation of PFKP at Ser386 by AKT promotes glycolysis and brain tumor growth [43].

Intriguing evidence indicates that pyruvate kinase (PK), which catalyzes the ATP-producing third rate-limiting step of glycolysis, plays a major role in controlling the balance between ATP production and biological precursors for malignant cell growth [15,44]. Four main isoforms of pyruvate kinase are found in mammalian cells: PKM1, PKM2, PKR, and PKL [45]. The isoform PKM2 impacts various anabolic events in cancer [45]. In cancer cells, the expression of PKM2 is higher than that of PKM1 [46], the latter which is a constitutively active tetramer [45]. PKM2 has been reported to provide growth advantages for cancer cells [47]. Serine and F1,6-BP are two activators of PKM2 [15]. PKM2 exists as a low-activity catalytic dimer or highly active tetramer [48], and a balance of these two forms has been shown to be important for tumorigenesis [47]. PKM2 has been identified to play a significant role in leukemogenesis [49,50]. In leukemia cells, SUMOylation of PKM2 results in the destabilization of PKM2 tetramer [51]. PGAM1 (phosphoglycerate mutase 1) catalyzes the conversion of 3PG to 2PG during glycolysis [14]. Phosphoenolpyruvate (PEP), the substrate for pyruvate kinase, is involved in the phosphorylation of PGAM1. Cancer cells with reduced PKM2 activity indicated the availability of an alternative pathway where PEP is converted to pyruvate through phosphorylation of PGAM1 by PEP, in which PEP acts as a phosphate donor [52]. PKM2 is closely associated with aggressive clinical features of liver cancer [53]. The activity of PKM2 is controlled by cellular antioxidant events. Elevated reactive oxygen species (ROS) inhibit the activity of PKM2 through the oxidation of Cys (358) residue, divert glucose into the PPP, and trigger redox balance, thereby supporting cancer cell survival under oxidative stress under continued antioxidant responses. The replacement of Cys (358) with a non-oxidizable mutant (Ser(358)) or PMK2 activators enhanced the sensitivity to oxidative stress and reduced tumor growth by reducing the flux into the PPP, rationalizing the inclusion of PKM2 activators as ideal therapeutics in combination with oxidative stress, thereby promoting therapeutics for cancer [54]. 

In the last step of glycolysis, lactate dehydrogenase (LDH) catalyzes the reversible conversion of pyruvate to lactate. LDHs are tetrameric enzymes encoded by four different genes: *LDHA*, *LDHB*, *LDHC,* and *LDHD* [55]. The isozymes LDHA and LDHB are highly expressed and alternate between homotetramers and heterotetramers [56]. LDHA, which is encoded by the c-Myc-responsive gene [57], is largely found in cancer and is required to maintain an increased glycolytic flux [57]. It is associated with poor prognosis of tumors [58]. When lactate dehydrogenases catalyze the reversible conversion of pyruvate to lactate, NAD^+^ is generated from NADH. This is necessary to maintain continued pyruvate to lactate flux, as a stock of NAD^+^ is necessary as a cofactor for glyceraldehyde 3-phosphate dehydrogenase (GAPDH) [15]. Since lactate inhibits the activity of PFK1, its intracellular accumulation should be barred to avoid the inhibition of the second committed step of glycolysis. Lactate provides proliferative advantages for cancer cells [59]. The knockdown of LDHA results in the inhibition of tumor propagation [60,61]. It is interesting to note that oxygenated tumors can uptake lactate generated by hypoxic tumor cells [62]. MYC and HIF1/2α have been reported to increase the expression of LDHA [63,64]. 

The intermediates of glycolysis, the PPP, and the TCA cycle serve as building blocks for the synthesis of amino acids. Amino acids serine, glycine, and cysteine derive from 3PG. In the first step of the serine biosynthesis pathway, the conversion of 3PG to 3-phosphohydroxypyruvate is catalyzed by phosphoglycerate dehydrogenase (PHGDH), where 3PG undergoes oxidation using NAD^+^. In the next step, phosphoserine aminotransferase catalyzes the conversion of 3-phosphohydroxypyruvate to p-phosphoserine. Serine serves as the precursor of glycine. Through a reversible reaction catalyzed by serine hydroxymethyltransferase (SHMT), serine is converted to glycine following the removal of a carbon atom. In this reaction, tetrahydrofolate (THF) accepts the β-carbon of serine to yield N^5^,N^10^-methylenetetrahydrofolate. Many types of tetrahydrofolates serve as one-carbon unit donors in critical metabolic reactions [14]. Glycine degradation through the mitochondrial glycine cleavage system gives one-carbon units required for the tetrahydrofolate (THF) cycle [14,65]. Serine and glycine are thus indispensable for nucleotide synthesis, protein synthesis, phosphatidyl-serine, and sphingosine synthesis [66]. 

Serine-biosynthesis pathway plays a key role in cancer, and high PHGDH expression maintains a high serine synthesis flux [67,68,69]. On the contrary, excess dietary intake of glycine demonstrates in vivo tumor inhibitory potential [70]. PHGDH inhibition by a small molecule containing piperazine-1-carbothioamide scaffold resulted in the conversion of glycine back to serine by the action of cytosolic serine hydroxymethyl transferase (SHMT1) [71]. In lung cancer cells, SHMT1 inhibition is not due to serine or glycine starvation, indicating different roles of SHMT1 other than the biosynthesis of serine from 3-phosphoglycerate [72]. With the assistance of modern technologies, folate-dependent NADPH generation has been identified as an important pathway for generating one-carbon units for nucleic acid synthesis and reducing power for cancer cells [73,74]. Glutathione, one of the key cellular antioxidants, is synthesized from glycine together with two other amino acids cysteine and glutamate. Glutathione metabolism shows pathogenic and preventive roles in tumorigenesis [75]. A recent study shows the role of glycine in maintaining glutathione in multiple myeloma. Glycine deprivation caused the inhibition of myeloma cell proliferation by disturbing the glutathione balance [76]. 

**Figure 3:**
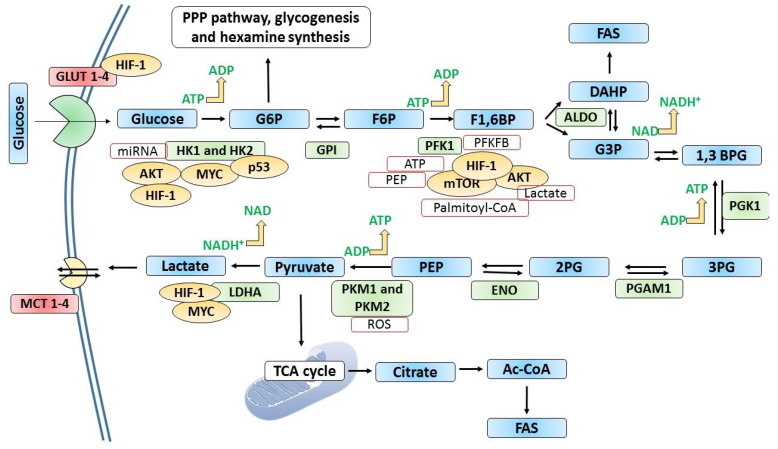
The metabolism of glucose in cancer cells. In many cancer types, glucose uptake and glycolysis occur at faster rates compared to normal cells. When the oxygen supply or levels are scarce, pyruvate generated by glycolysis is diverted away from mitochondrial oxidative phosphorylation by producing lactate (anaerobic glycolysis). Otto Warburg observed that glycolysis occurs at a higher rate in cancer cells despite the presence of oxygen, converting more glucose to lactate. This “Warburg” effect is the fundamental basis for detecting and treating several human tumors. For example, the phosphorylated form of [18F]2-fluoro-2-deoxy glucose (FdG) is not converted in the GPI-catalyzed reaction in glycolysis, where its accumulation is detected using positron emission tomography. Glucose transporters and many glycolytic enzymes are overexpressed or overproduced in cancer cells. Hypoxia-inducible factor-1 (HIF-1) induces or regulates the expression of key glycolytic enzymes and GLUTs. GLUT1 and GLUT3 are frequently overexpressed in cancer. Tumor suppressor p53 and some oncoproteins such as AKT, MYC, and mTOR regulate the expression of key glycolytic enzymes as shown in the figure. In addition, metabolites such as ROS, ATP, PEP, lactate, and palmitoyl-CoA exert regulatory effects as indicated. 1,3 BPG; 1,3-bisphosphoglycerate, 2PG; 2-phosphoglycerate, 3PG; 2-phosphoglycerate, ALDO; aldolase, DAHP; dihydroxyacetone phosphate, ENO; enolase, F1,6BP; fructose-1,6-bisphosphate, F6P; fructose-6-phosphate, FAS; fatty acid synthesis, G3P; glyceraldehyde 3-phosphate, G6P; glucose 6 phosphate, GLUTs; glucose transporters, GPI; glucose-6-phosphate isomerase, HK1 and HK2; hexokinase 1 and 2; LDHA; lactate dehydrogenase, MCT; monocarboxylate transporters, PEP; phosphoenolpyruvate, PFK1; phosphofructokinase-1, PFKFB; 6-phosphofructo-2-kinase/fructose-2,6-bisphosphatase, PGAM1; phosphoglycerate mutase 1, PGK1; phosphoglycerate kinase 1, PKM1; pyruvate kinase 1 and PKM2; pyruvate kinase 2.

Apart from committed reactions, non-committed reactions of glycolysis also drive tumorigenesis and play essential roles in cancer [77,78]. For example, the enzyme glucose -6-phosphate isomerase (GPI) catalyzes the inter-conversion of glucose-6-phosphate and fructose-6-phosphate. Outside of cells, this enzyme can function as a cytokine or growth factor [79]. The expression of GPI is dysregulated in a wide range of human cancers and is associated with poor prognosis [80,81]. Breast cancer stem cells secrete GPI and promote stem-like phenotype [82]. The molecular interaction of GAPDH with the spliced forms of GPI and PKM2 has been identified as one of the possible reasons for the elevated glycolytic activity in cancer cells [83]. Figure 3 shows anaerobic metabolism of glucose in a cancer cell. 

## 4. Impact of Glucose Metabolism on Cancer Hallmarks

### 4.1. Sustained Proliferation 

Sustained proliferation is one of the hallmarks of cancer [84]. Glucose accelerates the growth of cancer cells, and glucose deprivation leads to cell death in cancer cells, which is associated with reduced redox potential [85,86,87]. A number of epidemiological studies have also identified hyperglycemia as a risk factor for cancer [88,89,90]. A recent investigation demonstrates that glycemic variability (GV) and obesity are associated with an increased risk of developing cancer [88]. In a Swedish population, diabetes and impaired glucose metabolism were associated with an increased risk of postmenopausal breast cancer and endometrial cancer, respectively [89]. In addition, hyperglycemia has been reported to increase tumor volume and growth in vivo [91].

Hyperglycemia induced by glucagon was found to induce tumor growth and angiogenesis through a HIF/VEGF-associated signaling pathway [92]. Hyperglycemia was found to disrupt the function of HIF-1 inhibitors (berberine and HIF-1 small interfering RNA (siRNA)), which confirmed the involvement of HIF-1 associated signaling pathway in glucagon-induced hyperglycemia [92]. In an investigation conducted by Li et al. in 2018, a novel molecular mechanism between miR-301a and hyperglycemia was established in prostate cancer growth [93]. In prostate cancer cells, hyperglycemia or treatment with high glucose concentrations induced the expression of miR-301 and promoted cell proliferation of pancreatic cancer cells in a nude mouse model by repressing Smad4 and p21 [93]. 

The proliferation of MCF-10A mammary epithelial cells and MCF-7 hormone receptor-positive breast cancer cells was enhanced by hyperglycemia through the activation of AKT/mTOR and leptin/IGF1R signaling [94]. The effects of high glucose concentrations on epithelial–mesenchymal transitions (EMT) in bladder cancer have been investigated [95]. High glucose levels promoted the proliferation and invasion of bladder cancer cells in vitro and in vivo. The key components of the Hippo signaling pathway, YAP1 and TAZ, were identified as the key targets of high-glucose-induced EMT in bladder cancer [95]. Higher glucose concentration increases the aggressiveness of triple-negative breast cancer cells that was accompanied by increased cell migration, induced EMT, and decreased nuclear chromatin condensation [96]. A dose-dependent cell proliferation was observed in ovarian cancer cells exposed to glucose with increased accumulation of ATP and lactate levels [97]. In endometrial cancer cells, high glucose significantly increased the proliferation through modulating AMPK/mTOR/S6 and MAPK signaling, while low glucose exposure induced apoptosis [98]. 

The pro-tumoral effects of glucose and its effects on the expression of angiotensinogen in breast cancer cells were investigated by Sun et al. in 2019 [99]. High glucose was found to support the growth of breast cancer cells by promoting cell migration and invasion. High glucose exposure suppressed the expression of angiotensinogen, which was completely reversed with low glucose exposure, indicating that angiotensinogen expression is subjected to a negative regulation action of glucose in breast cancer cells. Overexpression of angiotensinogen reversed high glucose–induced proliferative and metastatic properties of breast cancer cells, highlighting how angiotensinogen can regulate the high glucose exposure in breast cancer cells [99]. It may be hypothesized that the vasoconstriction brought by angiotensin, restricts glucose availability to the tissues, which in turn may hinder cell proliferation.

Epidermal growth factor receptor (EGFR) plays a key role in mitogenic signaling [100]. Overexpression of hyperactivity of EGFR is seen in a range of human cancers and it is considered a promising anticancer target [101]. Rho family GTPases, such as Cdc42, RhoA, and Rac1, act as molecular bridges in mitogenic signaling cascades [102]. High glucose promoted the proliferation of breast cancer cells through the induction of EGFR activation (EGFR phosphorylation) accompanied by the activation of Rac1 and Cdc42, implying the necessity of Rac1 and Cdc42 in the activation of EGFR following high glucose exposure. Notably, Cdc42 stimulated EGFR phosphorylation by preventing EGFR degradation by Cbl proteins, a family of ubiquitin ligases (E3s). In contrast, Rac1-mediated EGFR activation is independent of EGFR degradation [103]. Interestingly, a mathematical prediction tool has been developed to predict how glucose availability and consumption influence the proliferation and death of cancer cells in vitro [104]. 

Protein kinase C-delta (PKCδ) is sensitive to cellular metabolic alterations and plays a key role in the regulation of cell proliferation and apoptosis [105]. Elevated glucose metabolism was found to inhibit PKCδ-mediated activation of p53 to sustain cell proliferation following growth factor removal. On the other hand, p53 activation due to DNA damage was independent of PKCδ [106]. In addition, increased glucose metabolism was found to confer resistance to imatinib treatment, a BCR-ABL kinase inhibitor, providing a bridge between glucose metabolism and drug resistance and uncovering novel pharmacological targets implicated in tumor glucose metabolism [106].

### 4.2. Apoptotic Resistance

Cancer cells override apoptosis, thereby gaining excessive proliferation [107]. Intriguing evidence indicates that reprogrammed glucose metabolism influences signaling pathways associated with apoptosis [108,109]. The serine/threonine kinase Akt/PKB is a key determinant of cell survival and proliferation. AKT has been reported to enhance glycolysis and lactate generation [15,110]. In cancer cells, the activity of AKT helps to determine the aerobic glycolysis shift [110]. High rates of aerobic glycolysis were evident in human glioblastoma cells with an activated form of AKT [110]. AKT has been reported to mediate the expression of the GLUT1 gene, the most widely expressed glucose transporter in mammalian cells [111].

Evidence indicates that AKT exerts anti-apoptotic effects triggered by various apoptotic stimuli [112]. The release of cytochrome C from mitochondria and the activation of pre-apoptotic BCl-2 family proteins such as BAX are events in the apoptotic cascade leading to loss of mitochondrial membrane integrity. AKT has been reported to prevent both cytochrome c release from the mitochondria [113] as well as the activation of BAX [114,115] in mechanisms linked to the regulation of glucose metabolism, thereby preventing the progression of apoptosis. AKT helps to maintain the integrity and function of mitochondria through the availability of glucose and its metabolism [114]. The availability of glucose and its metabolism are critical for the inhibition of cytochrome c release from the mitochondria. However, it was shown that glucose phosphorylation, the first committed glycolytic event catalyzed by hexokinase, was sufficient for AKT to promote cell survival by inhibiting apoptosis [114]. 

This is in line with the evidence that overexpression of HK2 in cancer cells confers resistance to apoptosis [116]. A considerable fraction of cellular HK2 is located in mitochondria through docking with the voltage dependent anion channel (VDAC), which allows the movement of small molecules across the outer mitochondrial membrane [117]. The interaction of HK2 and mitochondria is triggered by AKT in the presence or absence of Bax/BaK [118]. HK2 detachment from mitochondria induced apoptosis through phosphorylation of VDAC mediated by GSK3β, indicating that HK2 possesses some extra characteristics relevant to apoptosis [119,120]. 

The influence of ATP for caspase-dependent apoptosis in cancer cells is well-described [121,122]. Caspase-dependent apoptosis induced in HeLa cells was found to be associated with reduced cellular ATP levels, suggesting that caspases can block the activity of cellular survival mechanisms. The inhibition of cellular ATP contents observed in HeLa cells was correlated with the caspase-dependent inhibition of glycolysis. The activity of rate-limiting enzymes phosphofructokinase and pyruvate kinase was impaired by caspases, whereas the activity of phosphoglycerate kinase was not altered by caspases, indicating specificity in the caspase inhibition of glycolytic enzymes. In addition, caspases did not affect the TCA cycle, PPP, and glutamine pathways, demonstrating that caspases affect metabolic pathways associated with glucose metabolism in a selective manner [123]. 

The induction of PUMA, a pro-apoptotic Bcl-2 family protein, by p53 is metabolically controlled by glucose or growth factor withdrawal, and glucose promotes anti-apoptotic signaling cascades to diminish apoptosis induced by growth factor withdrawal through the inhibition of GSK to suppress the activation of fifty3 and p53 dependent induction of pro-apoptotic PUMA, giving anti-apoptotic support that aids cell-survival [124]. 

Interestingly, AKT phosphorylates and suppresses the proapoptotic properties of Foxo1 and Foxo3a [125] and BAD [126]. Moreover, AKT phosphorylates HK2 at Thr-473 and reduces its dissociation from the mitochondria, leading to protection from apoptosis [30]. The overexpression of induced myeloid leukemia cell differentiation protein (Mcl-1), a member of anti-apoptotic Bcl-2 family proteins, is seen in a range of human cancers [127,128]. The association of Mcl-1 in chemo and radiotherapy resistance in cancer cells is well-described [129]. The function of Mcl-1 is closely linked with the resistance to ABT-737, a Bcl-2 family inhibitor [130]. Furthermore, Mcl-1 is associated with various cellular metabolic activities [131,132,133]. 

Glucose metabolism is indispensable to sustain Mcl-1 protein synthesis, and the inhibition of glucose metabolism results in a reduction of Mcl-1 levels, which helps to overcome Bcl-2 family inhibitor resistance, rationalizing the use of glucose metabolism targeted therapies in sensitizing cancer cells to apoptosis through the suppression of the expression of Mcl-1 [134]. The kinesin family member C1 (KIFC1) is overexpressed in a range of human cancers and its upregulation is correlated with poor prognosis [135]. A recent investigation demonstrates that KIFC1 promotes aerobic glycolysis through the HMGA1/c-myc pathway, and the inhibition of KIFC1 promoted the inhibition of endometrial cancer cell growth [136]. A review by Matsuura et al. in 2016 illustrates the role of metabolic regulation in apoptosis and how metabolic regulation affects key signaling components linked to apoptosis [137]. 

### 4.3. Angiogenesis 

Angiogenesis is a hallmark feature of cancer [138]. Glucose metabolism plays an essential role in tumor angiogenesis. Tumor endothelial cells demonstrate an enhanced glycolytic rate and increased activity of the oxidative pentose phosphate pathway among others in order to sustain the proliferation [139]. This hyper-glycolytic state is likely to be induced by the hypoxia inducible factor (HIF) in response to the harsh and hypoxic conditions in the microenvironment of the tumor endothelial cells. Furthermore, HIF-1 plays a key role in the regulation of angiogenic factors including VEGF and VEGFR. Overexpressed HIF-1 in human cancers has been reported to be associated with vascularization [140,141]. As a transcription factor, HIF-1 is involved in the regulation of the expression of genes associated with angiogenesis. [142]. The ectopic expression of HIF-1α in p53-deficient colon cancer cells resulted in increased expression of VEGF-enhanced angiogenesis and tumor growth, highlighting the importance of HIF-1 through the loss of function of p53 during tumorigenesis. This rationalizes HIF-1 as an ideal pharmacological target for p53-deficient cancers [142]. 

The proto-oncogene *c-Myc* is a multi-purpose proto-oncogene that is often dysregulated in human cancers [143]. HIF-1 was found to work cooperatively with dysregulated c-Myc to promote tumorigenesis by enhancing glycolysis [144]. Enhanced glycolysis by the synergistic action of c-Myc and HIF-1 was associated with induced expression of HK2 and pyruvate dehydrogenase kinase 1, the latter that stops coupling of glycolysis to mitochondrial glucose oxidation by inhibiting pyruvate dehydrogenase. In addition, the combined action of c-Myc and HIF-1 contributed to tumorigenesis by inducing the expression of angiogenic factor VEGF at m-RNA and protein levels [144]. 

In hepatocellular carcinoma, a significant correlation was observed between HK2 mRNA and HIF-Iα protein levels. Contrastingly, the association between a higher expression of HIF-1α protein and VEGF mRNA levels was not significant in metastatic liver cancer [145]. A significant association between the expression of HIF1α and HIF2α and angiogenic factors (VEGF, PD-ECGF and, bFGF) was evident in non-small-cell lung cancer cells [146]. The co-expression of VEGF and HIF-I was observed in Wilms tumors (nephroblastoma) [147]. 

Ryan et al. in 1998 demonstrated the essential roles of HIF-1 in embryonic vascularization and solid tumor formation in vivo [148]. The enzyme PFKFB3 is considered a key regulator of glycolysis [34,149]. Recent investigations address the glycolytic roles of PFKFB3 in cancer [34,35,36,149]. PFKFB3 suppression by 3-(3-pyridinyl)-1-(4-pyridinyl)-2-propen-1-one (3PO) resulted in the inhibition of glycolysis and impaired vessel sprouting, delayed formation of capillary tubes and promoted the anti-angiogenic efficacy of VEGFR inhibitors, suggesting a PFKFB3-linked therapeutic window to suppress angiogenesis [150]. In an attempt to investigate the role of PFKFB3 in angiogenesis, the inhibition or deletion of PFKFB3 suppressed angiogenesis both in vitro and in vivo, and AKT was found to be a mediator of PFKFB3 [151]. Sorafenib is a well-established antiangiogenic agent [152]. Hypoxia reduced the therapeutic effects of sorafenib, while HK2 inhibition re-established its therapeutic potential [153]. Pericytes, also known as mural cells, found in the basement layer of blood microvessels are involved in key pathological and physiological processes, including blood vessel formation [154]. Pericytes support angiogenesis and increase the clinical efficacy of anti-cancer agents by modulating the tumor microenvironment (TME) [155]. 

In a recent investigation, Meng et al. in 2021 demonstrated a different approach that can be used to remodel tumor vasculature by manipulating the metabolic profile of pericytes derived from tumors [156]. HK2 was found to drive glycolysis in tumor-derived pericytes and regulate the blood vessel supporting ability of pericytes by inducing Rho-associated coiled-coil containing protein kinase 2 -myosin light chain 2 (ROCK2-MLC-2) driven contractility. HK2 inhibition in tumor-derived pericytes supported the blood vessel formation ability and improved the efficacy of doxorubicin against tumor formation [156]. In melanoma cells, HK2 regulated angiogenesis through p38-MAPK signaling [157]. 

The protein disulfide isomerase (PDI) family belongs to a group of ER enzymes, which mediates key functions in the re-arrangement of protein disulfide bonds [158]. PDI activity is involved in almost every vital step of tumorigenesis, making PDI a promising therapeutic target [159]. The combined exposure of HK2 (3-bromopyruvate) and PDI inhibitors (bacitracin) resulted in the reduction of microvessel densities, (a surrogate marker for tumor angiogenesis), in an in vivo model of hepatocellular carcinoma [160]. Elevated lactate production is an ideal indicator of the metabolic adaptation of tumors and is associated with clinical outcomes in human cancers [161]. Glucose-deprived cancer cells utilize lactate as a fuel [162]. Lactate-induced angiogenic neovascularization in cancer cells through the activation of NF-kB/IL-8 (CXCL8) signaling in endothelial cells [163]. 

### 4.4. Metastasis 

Metabolic alterations and accelerated glycolysis are common in metastatic tumors [164,165]. Increased expression of HIF-1α is predictive of cancer metastasis [166]. HIF-1α promotes metastasis in a range of human cancers [167,168,169]. In addition to HIF-1α, the metastatic role of HIF-3α has been shown in pancreatic cancer, indicating the involvement of different HIF-α subunits in metastasis [170]. In breast cancer cells, the inhibition of HIF (HIF-1α and HIF-2α) resulted in the inhibition of breast cancer tumor growth and lung metastasis by suppressing the expression of angiopoietin-like 4 (ANGPTL4) and L1 cell adhesion molecule (L1CAM) [171]. HK2 regulated tumorigenesis and metastasis by directly regulating glycolysis in pancreatic ductal adenocarcinoma [172]. A recent investigation identified that increased expression of HK2 correlates with the metastatic ability of breast cancer [173]. 

The histone variant H2AX plays a prominent role in DNA damage response by recruiting DNA damage repair proteins to double stranded break (DSB) damage [174]. H2AX has been identified as an important activator of key transcription factors implicated in epithelial–mesenchymal transition (EMT) [174]. 

An investigation by Liu et al. in 2022 demonstrated the role of histone H2AX in breast cancer metastasis through HK II-mediated glycolysis [175]. Recent studies have identified the role of glucose-6-phosphate dehydrogenase (G6PD) in metastasis. G6PD, the rate-limiting enzyme of the PPP pathway, is up-regulated in omental metastases, and pharmacological suppression of G6PD resulted in the inhibition of omental metastases [176]. Ectopic expression of G6PD augments cellular antioxidant mechanisms, leading to oncogenic transformation in vitro and in vivo [176], which indicates the necessity of G6PD for maintaining cellular antioxidant events. A recent investigation demonstrates that G6PD is not indispensable for oncogenic K-Ras driven tumors [177]

Healthy antioxidant capacity has been reported to trigger cancer cell survival under various stress conditions [178]. Under various stress conditions, pancreatic cancer cells show increased de novo NADP+ synthesis through the up-regulation of G6PD to increase the efficacy of cellular antioxidant mechanisms. The expression of G6PD and its upstream activators (NRF2, E2F1, and TAp73) is higher in metastatic pancreatic cancer. G6PD knockdown prevented pancreatic cancer metastasis, highlighting the importance of G6PD-associated NADP+ generation associated with pancreatic cancer cell proliferation and metastasis [179]. PKM2 was found to promote metastasis in hepatocellular carcinoma cells indicating an oncogenic function of PKM2 [180]. Jin et al. in 2017 illustrated LDHA phosphorylation and activation-mediated metastatic properties [181].

## 5. Epigenetic Impact on the Glycolytic Events 

Epigenetic events are indispensable for the maintenance of tissue-specific gene expression patterns. In addition to dysregulated metabolic events, dysregulated epigenetic events are frequently observed in cancer cells, which have diverse effects on the hallmarks of cancer, depending on cancer types and grades [182]. In cancer cells, dysregulated epigenetic events and various genetic abnormalities result in the inactivation of tumor suppressor genes and aberrant activation of signaling pathways, working together to promote various events in tumorigenesis [182]. The precise patterns of DNA methylation, histone methylation, histone acetylation, and histone phosphorylation as well as the activity and expression of chromatin modifying enzymes observed in normal cells are distorted in cancer cells, serving as driving forces to promote tumorigenesis [183]. Unlike genetic alterations, altered epigenetic events are reversible, making epigenetics-based therapies promising pharmacological approaches while challenging the traditional view and beliefs of the altered genetic events being the key factor for many diseases. It is interesting to note that the metabolic adaptations in cancer cells are connected at the epigenetic level [184].

A recent investigation shows that vorinostat, a well-established histone deacetylase (HDAC) inhibitor, re-programs glycolytic events in neuroblastoma (NB) cells [185], implying that HDACi can influence metabolic events in cancer cells. The kinetic properties of chromatin-modifying enzymes, for example, histone deacetylases and DNA methyltransferases, are controlled by several exogenous and endogenous metabolic substrates [184]. Pyruvate (PYR) generated in glycolysis is converted to Acetyl-Co-EnzymeA (Ac-CoA), which is catalyzed by pyruvate dehydrogenase (PDH). Ac-CoA is also generated by fatty acid β-oxidation, catabolism of some amino acids, degradation of ketone bodies, and oxidative decarboxylation of pyruvate. The concentration of cellular Ac-CoA affects histone acetylation in various cell types [186,187].

The acetyl-CoA levels are regulated by glucose availability and the acetyl-CoA: coenzyme A ratio is one of the determinants of histone acetylation levels in cancer cells. KRAS mutations drive acetyl-CoA generation and affect histone acetylation through an AKT-dependent molecular mechanism, which is mediated through the activity of ATP-citrate lyase [188]. One-carbon (1C) metabolism, which is centered on folate and methionine cycles, produces 1C units (methyl groups) required for the production of nucleotides, thymidylate synthesis, and purine synthesis. The methionine provides S-adenosylmethionine (SAM), which serves as a substrate for methylating histone proteins. In posterior fossa tumors, hypoxia-induced metabolic re-programming is accompanied by reduced SAM levels and increased α-KG and acetyl-CoA levels, which influenced H3K27 hypomethylation [189]. 

Mutations in the NADP+-dependent isocitrate dehydrogenase genes *IDH1* and *IDH2* are common in acute myeloid leukemia and gliomas [190,191]. IDH1 and IDH2 mutations result in the production of reduced alpha-ketoglutarate (α-KG) levels and increased 2-hydroxyglutarate (2-HG), which influence histone and DNA methylation profiles in glioma [192].

JmjC domain-containing histone demethylase 1 (JHDM1) specifically demethylates histone H3 at lysine 36 (H3-K36) using alpha-ketoglutarate α-KG as a co-substrate [193]. Epstein-Barr virus (EBV) is an oncogenic virus and accounts for nearly 1% of all human cancers [194]. Latent membrane protein 1 (LMP1) is indispensable for EBV-mediated malignant transformation [195]. The ectopic expression of LMP1 in nasopharyngeal carcinoma cells promoted glycolysis via the upregulation of HK-II [196]. Another study by the same group demonstrated the involvement of DNMT1 in aerobic glycolysis and LMP1 triggers localization of DNMT1 to inhibit OXPHOS complex gene expression [197]. A recent investigation addresses the role of HDAC6 in regulating glycolytic metabolism [198]. The inhibition of HDAC6 in triple-negative breast cancer cells showed reduced levels of glycolytic metabolites such as PEP, 3-phosphoglyceric acid (3PG), lactate, and pyruvate [198], confirming the involvement of HDAC6 in regulating glycolysis in breast cancer cells. 

## 6. Pharmacological Targets 

Intervention of the reprogrammed glucose metabolism in cancer progression, by slowing down or inhibiting the accelerated glucose metabolism through the suppression of major glucose metabolism enzymes, has been shown as a promising antitumor strategy. Over the past decades, pre-clinical and clinical drug discovery approaches have identified natural and synthetic drug candidates as glucose-metabolism-targeting drugs. HKs (HK1 and HK2), PFK1 and PFK2, G6PD, pyruvate kinase, phosphoglycerate dehydrogenase (PHGDH), lactate dehydrogenase, phosphoglycerate kinase 1 (PGK1), enolase (ENO), isocitrate dehydrogenase (IDH), and glucose transferase (GLUT) serve as ideal pharmacological targets to treat cancer. 2-deoxyglucose (2-DG), 3-bromopyruvate (3-BrPA), and metformin (Met) are well-known HK2 enzyme inhibitors [199]. 3-bromopyruvate (3-BrPA) exerts anti-cancer effects by targeting HK2 [200,201]. 2-DG also demonstrates a wide range of anti-cancer effects through the inhibition of HK2 activity [202]. Benserazide, a drug used to treat Parkinson’s disease, selectively inhibited the activity of HK2 and induced apoptosis in colorectal cancer cells [203]. Benitrobenrazide was identified as a sub-micromolar potent HK2 inhibitor [204]. Inhibition of the activity of HK2 enzyme by arsenic is a key mediator of arsenic-induced apoptosis in cancer cells [205]. 

The combined treatment of D-mannoheptulose, a hexokinase inhibitor, and the oncolytic virus NDV was identified as an ideal strategy for breast cancer cells [206]. This combination selectively inhibited glycolytic products in breast cancer cells without affecting normal cells [206]. Furthermore, well-known secondary metabolites such as berberine, epigallocatechin gallate, and quercetin mediate their anticancer effects through the inhibition of the activity of HK2 [207,208]. 

Resveratrol inhibits breast cancer cell proliferation by inhibiting 6-phosphofructo-1-kinase activity [209]. Acetylsalicylic acid and salicylic mediate anticancer activity in breast cancer cells through the inhibition of 6-phosphofructo-1-kinase (PFK1) activity [210]. Clotrimazole is an anti-fungal agent. It is a promising anti-tumor agent [211]. Clotrimazole modulated glycolytic flux by inhibiting the activity of PFK1 [211]. A derivative of the small molecule 3-(3-pyridinyl)-1-(4-pyridinyl)-2-propen-1-one (3PO) exhibited PFKFB3 inhibitory potential with promising anti-cancer effects [212]. PFK15, a derivative of 3PO, is a potent inhibitor of PFKFB3. PFKFB3 is an allosteric activator of PFK1 [15]. Combined exposure of PFK15 and metformin displayed anti-myeloma effects through the PFKFB3/MAPKs/STAT signaling pathway [213]. A recent investigation demonstrates that combined treatment of PFK-015 and PD-1 monoclonal antibody suppressed tumor formation in vivo [214]. PFK158, a structural analog of PFK-015, is also a PFKFB3 inhibitor that can exert anti-cancer effects [215]. 

Polydatin, a resveratrol derivative [216], is a glucose-6-phosphate dehydrogenase (G6PD) inhibitor. Polydatin exerted anti-tumor effects in vivo [217]. Hong et al. in 2018 demonstrated that G6PD inhibition by siRNA can sensitize lung cancer cells to cisplatin by influencing redox homeostasis [218]. The exposure of colorectal cancer cells to aspirin resulted in elevated acetylation of G6PD, which decreased its activity, contributing to the anti-cancer effects of aspirin [219]. Dehydroepiandrosterone (DHEA) is a steroid hormone precursor [220]. The inhibition of G6PD activity by DHEA resulted in the induction of apoptosis in cervical cancer cells [220]. RRx-001 is an aerospace compound that belongs to the class of dinitroazetidines. RRx-001 demonstrated anticancer effects by inhibiting the activity of G6PD [221]. In addition to small molecules, several plant extracts have also been identified to have anti-cancer properties that are accompanied by the inhibition of the activity of G6PD [222,223]. 

The inhibition of the LDH activity in cancer cells by the small molecule termed FX11 was identified as a therapeutic approach to the Warburg effect [224]. Lactate dehydrogenase A inhibitors NHI1 and NHI2 displayed synergistic anticancer effects with gemcitabine [225]. PSTMB, a small molecule, suppressed the activity of LDHA and lactate production in cancer cells [226]. The anticancer effects of an LDHA inhibitor, galloflavin, a gallic acid derivative, have been reported in endometrial cancer cells [227]. In an attempt to identify LDHA inhibitors, 24c, a pyrazole-based small molecule was identified as a potent LDHA inhibitor with the anti-proliferative ability [228].

Monocarboxylate transporters (MCTs) are plasma membrane transport proteins that serve as carriers for lactate, pyruvate, short-chain fatty acids (propionate and butyrate), and ketone bodies to the extracellular space [229]. There are 14 members in the MCT family (MCTs 1–14) and some members are ubiquitously expressed [230]. 

In glycolytic tumors, MCTs 1–4 aid the movement of lactate, which is found abundantly, thereby helping to maintain appropriate pH and avoid intercellular acidification [230]. AZD3965, a pyrimidinedione, 3-carboxy coumarins, and indol-3-yl-cyanoacrylic acids have been identified as small-molecular MCT inhibitors with anticancer activities [231]. A recent investigation identified fluvastatin as an inhibitor of MCT4 for lung cancer [232]. 

Targeting GLUT1 and GLUT3, which are frequently overexpressed in cancer cells, has been identified as a promising anti-cancer strategy [233,234]. Several natural and synthetic GLUT1 inhibitors with anticancer potential have been identified. For example, natural compounds with known anti-proliferative potentials such as genistein, quercetin, resveratrol and curcumin are GLUT1 inhibitors [235,236,237,238]. The small molecule SMI277 was identified as a novel GLUT1 inhibitor recently. SMI277 inhibited glucose uptake, decreased lactate secretion, and induced apoptosis in tumor cells [239]. Siebeneicher et al. in 2016 employed HTS to identify novel 1H-pyrazolo[3,4-d]pyrimidines as novel GLUT inhibitors. Some 1H-pyrazolo[3,4-d]pyrimidines derivatives exerted potent inhibitory effects toward GLUT1, GLUT2 and, GLUT3 at nano- and micro-molar IC_50_ fifty concentrations [240]. On the contrary, arachidonic acid, a polyunsaturated omega-6 fatty, functions as a GLUT1 and GLUT4 expression inducer in adipocytes [241].

In glycolysis and gluconeogenesis, the enzyme enolase (ENO) catalyzes the reversible conversion of D-2-phosphoglycerate (2PGA) and phosphoenolpyruvate (PEP) [14]. Overexpression of ENO is observed in human cancers. Compounds such as 2-phospho-D-glyceric, phosphonoacetohydroxamic acid, and sodium fluoride have been identified as enolase inhibitors [242]. Increased expression of enolase-alpha (ENO-1) mRNA was identified in estrogen receptor-positive tumors compared to ER-negative tumors. Inhibition of the expression of ENO-1 by siRNA enhanced the cytotoxic effects of tamoxifen in tamoxifen-resistant breast cancer cells, rationalizing the use of ENO-1 as a drug target to reverse tamoxifen resistance in breast cancer [243]. A small-molecule library screening identified AP-III-a4 as a potent inhibitor that displayed anti-cancer and anti-diabetes potential [244].

Phosphoglycerate mutase (PGM) catalyzes the transfer of carbon 3 to carbon 2 of phosphoglycerate [14]. MJE3, a spiroepoxide, blocks the activity of PGM and inhibits the proliferation of breast cancer cells [245]. Epigallocatechin-3-gallate, a well-known tea secondary metabolite, demonstrates PGAM1 inhibitory potential [246]. An anthraquinone derivative was found to be a potent PGM-1 inhibitor with in vivo anti-cancer effects [247].

**Figure 4:**
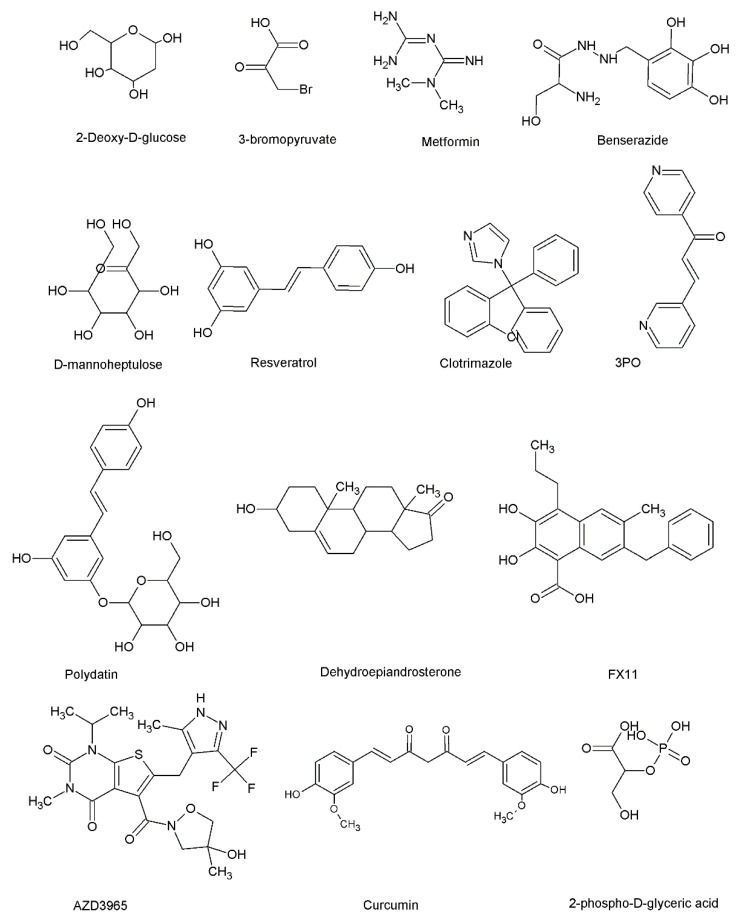
The chemical structures of some glycolytic enzyme inhibitors. 2-deoxyglucose, 3-bromopyruvate, metformin, benserazide, and D-mannoheptulose are HK inhibitors. Resveratrol, clotrimazole, and 3PO are PFK1 or PFKFB3 inhibitors. Polydatin and dehydroepiandrosterone are G6PD inhibitors. FX11 is an LDH inhibitor. AZD3965 is an MCT inhibitor. Curcumin is a well-known GLUT1 inhibitor. D-2-phosphoglyceric acid is an enolase inhibitor.

The small molecule TDZD-8 was found to possess anti-proliferative effects and aldolase inhibitory effects by reducing the stability of HIF-1α [248]. In lung cancer cells, aldolase A interacts with γ-actin to promote metastasis. Raltegravir, an antiretroviral drug, breaks aldolase A-γ-actin interactions to exert anti-metastasis effects in vitro and in vivo [249]. Glucose-6-phosphate isomerase acts as a cytokine and is associated with metastasis [250]. 6-phosphogluconate (6PG) and erytrose-4-phosphate (E4P) were identified as potent glucose-6-phosphate isomerase inhibitors with anti-breast cancer stem cell potential [82]. Figure 4 shows the chemical structures of some small molecules that inhibit the activity of glycolytic enzymes. Table 1 summarizes the anti-cancer effects of some of them.

## 7. Conclusions and Future Directions

Tumorigenesis is a complex and multi-step process. Various oncogenes and disrupted cellular signaling pathways have been reported to drive tumorigenesis. For example, the activity of major components of the phosphatidylinositol 3-kinase (PI3K)/AKT/mammalian target of rapamycin (mTOR) signaling pathway, a major signaling pathway responsible for cell proliferation and survival, are frequently deregulated in human cancers [251]. More recent investigations highlight the link between dysregulated signaling pathways and reprogrammed metabolic events in cancer cells, providing windows for therapeutic intervention in anti-cancer drug discovery [252]. Conventional metabolic events/programs are often dysregulated (suppressed or enhanced) in cancer cells as a result of irregularities in genetic and non-genetic or epigenetic factors. Altered metabolic pathways facilitate the proliferation and adaptation of cancer cells to stressful conditions by fulfilling bioenergetic and biosynthetic demands. Importantly, cancer cells fulfill their redox demands through altered metabolic events. Altered metabolic pathways are indispensable for metastasis [253]. Notably, altered metabolic events induce resistance to anti-cancer therapies [254].

Cancer cells display high glycolytic rates that offer proliferative advantages for cancer cells by providing glycolytic intermediates for subsidiary pathways that synthesize NADPH, nucleotides, amino acids, and lipids [255]. The hypoxia-induced factor 1 (HIF-1) is a key transcription factor that controls the expression of a number of genes associated with glycolytic flux in malignant cells [9]. HIF-1 supports cancer cells to depend on anaerobic metabolism. However, it is evident that hypoxia is not the key driving force that facilitates abnormal glycolytic flux in cancer cells as a wide range of genetic and epigenetic factors have been identified as HIF-1 activators. Moreover, it is interesting to note that some cancer cells like leukemic cells rely on glycolysis regardless of hypoxic conditions, implying that tumor hypoxia is not the key contributor that governs glycolytic switch in cancer cells [256].

Irregularities associated with metabolic events offer attractive therapeutic approaches for cancer. Since the activity of key glycolytic enzymes is altered or impaired, targeting such dysregulated glycolytic enzymes by using small molecules has been identified as an attractive approach. However, it is essential to assess whether targeting glucose metabolism can show meaningful clinical outcomes as normal cells and immune cells also possess glycolytic enzymes, necessitating the need of exploring metabolism-based anti-cancer therapies that can specifically target cancer cells. Investigations addressing reprogrammed glucose metabolism in cancer stem cells are limited. The identification of drug candidates against cancer stem cells is an emerging area in cancer drug discovery approaches. Therefore, investigators are encouraged to obtain a sharp metabolic picture of different cancer stem cell types to distinguish cancer stem cells from parental phenotypes metabolically. A recent investigation identified that breast cancer stem cells rely on glycolysis rather than oxidative phosphorylation (OXPHOS). Nootkatone, a natural small molecule, was found to impair glucose metabolism in breast cancer cells [257]. Furthermore, the application of medicinal chemistry is encouraged to identify novel small molecules that can target glucose metabolism focusing particularly on the isoenzymes that are dysregulated or having a critical role in cancer.

## Figures and Tables

**Table 1:** Small molecules that inhibit the activity of key glycolytic enzymes and their anti-cancer mechanism in brief.

	Anti-Cancer Mechanism/s Associated	Reference/s
**Hexokinase 2 inhibitor/s**		
2-deoxyglucose, 3-bromopyruvate, and metformin	2-deoxyglucose, 3-bromopyruvate, and metformin have been reported to exert anti-cancer effects through various molecular mechanisms in a range of cancer cells.	[199,200,201,202]
Benserazide	Selectively inhibits HK2 activity. Benserazide inhibited glycolysis, induced apoptosis, demonstrated inhibitory effects on the loss of mitochondrial membrane potential in cancer cells, and inhibited tumor formation in vivo.	[203]
Benitrobenrazide	Benitrobenrazide exerted HK2 inhibitory effects at nanomolar concentrations. Induced apoptosis in pancreatic cancer cells and inhibited tumor growth in vivo.	[204]
Arsenic	Arsenic directly binds with HK2 and induces apoptosis.	[205]
D-mannoheptulose	The combined treatment of Newcastle disease virus (NDV) and D-mannoheptulose showed synergistic anti-cancer effects and induced apoptosis.	[206]
**6-phosphofructo-1-kinase inhibitor/s**		
Resveratrol	Reduced the viability of breast cancer cells by disrupting glucose metabolism.	[209]
**PFK1 inhibitor/s**		
Acetylsalicylic acid and salicylic	Reduced the glucose consumption of breast cancer cells while exerting anti-proliferative effects in breast cancer cells.	[210]
Clotrimazole	Inhibition of mitochondrial-bound glycolytic enzymes, calcium-dependent potassium channel, and calmodulin.	[211]
**PFKFB3 inhibitor/s**		
PFK15 and metformin	Combined treatment of PFK15 and metformin demonstrated anti-myeloma effects through the PFKFB3/MAPKs/STAT signaling pathway	[213]
**G6PD inhibitor/s**		
Polydatin	Improves the accumulation of reactive oxygen species (ROS) and increases endoplasmic reticulum stress. Induces apoptosis and cell cycle block.	[217]
Aspirin	Acetylation of G6PD by aspirin contributes to anti-cancer effects.	[219]
**LDH inhibitor/s**		
FX11	Induces oxidative stress and inhibits tumor formation.	[224]
PSTMB	Exerted anti-proliferative effects and induced apoptosis in a range of cancer cells (liver, breast, colorectal, and lung).	[226]
**MCT inhibitor/s**		
AZD3965	Modulated tumor lactate levels and induced anti-tumor effects in vivo. The combined exposure of AZD3965 and inhibitors of glutaminase resulted in the induction of apoptosis in lymphoma cells.	[231]
**GLUT1 inhibitor/s**		
Genistein, quercetin, resveratrol, and curcumin	Exerts anti-cancer through various molecular mechanisms.	[235,236,237,238]
SMI277	SMI277 inhibited glucose uptake, decreased lactate secretion, induced apoptosis in tumor cells, and enhanced CD8+ T cell response.	[239]
**Phosphoglycerate mutase inhibitor/s**		
MJE3	Inhibits the proliferation of breast cancer cells	[245]
**Aldolase inhibitor/s**		
TDZD-8	Exerts anti-proliferative effects and aldolase inhibitory effects by reducing the stability of HIF-1α	[248]
Raltegravir	Breaks aldolase A-γ-actin interactions to exert anti-metastasis effects in vitro and in vivo.	[249]
